# Transients in the Palomar Observatory Sky Survey (POSS-I) may be associated with nuclear testing and reports of unidentified anomalous phenomena

**DOI:** 10.1038/s41598-025-21620-3

**Published:** 2025-10-20

**Authors:** Stephen Bruehl, Beatriz Villarroel

**Affiliations:** 1 Department of Anesthesiology, Vanderbilt University Medical Center, 701 Medical Arts Building, 1211 Twenty-First Avenue South, Nashville, TN 37212 USA; 2 Nordita, KTH Royal Institute of Technology and Stockholm University, Hannes Alfvéns Väg 12, 106 91, Stockholm, Sweden

**Keywords:** Transient, Unidentified anomalous phenomena, UAP, UFO, Nuclear testing, Transient astrophysical phenomena, Atmospheric chemistry

## Abstract

Transient star-like objects of unknown origin have been identified in the first Palomar Observatory Sky Survey (POSS-I) conducted prior to the first artificial satellite. We tested speculative hypotheses that some transients are related to nuclear weapons testing or unidentified anomalous phenomena (UAP) reports. A dataset comprising daily data (11/19/49—4/28/57) regarding identified transients, nuclear testing, and UAP reports was created (n = 2,718 days). Results revealed significant (*p* = .008) associations between nuclear testing and observed transients, with transients 45% more likely on dates within + /- 1 day of nuclear testing. For days on which at least one transient was identified, significant associations were noted between total number of transients and total number of independent UAP reports per date (*p* = 0.015). For every additional UAP reported on a given date, there was an 8.5% increase in number of transients identified. Small but significant (*p* = .008) associations between nuclear testing and number of UAP reports were also noted. Findings suggest associations beyond chance between occurrence of transients and both nuclear testing and UAP reports. These findings may help elucidate the nature of POSS-I transients and strengthen empirical support for the UAP phenomenon.

## Introduction

Transient star-like objects have been identified in sky surveys conducted prior to the launch of the first artificial satellite on October 4, 1957^1,2^. These short-lived transients (lasting less than one exposure time of 50 min) have point spread functions and are absent in images taken shortly before the transients appear and in all images from subsequent surveys^3^. As reported previously in this journal^3^, in some cases multiple transients appear in a single image, exhibiting characteristics not easily accounted for by prosaic explanations (e.g., gravitational lensing, gamma ray bursts, fragmenting asteroids, plate defects)^3,4^. We have identified numerous transients in the Palomar Observatory Sky Survey (POSS-I) as well as in other sky surveys as part of the Vanishing and Appearing Sources during a Century of Observations (VASCO) project^1–3^.

The source of the transients identified remains unknown and cannot be directly tested due to their historical nature. Nonetheless, examination of contemporaneous correlates of these transients may provide information useful for elucidating their possible origin. Systematic research of this type has not previously been conducted. However, anecdotal reports suggest speculative hypotheses regarding possible correlates of transients for which sufficient data are available to enable empirical testing.

Possible associations of transients with nuclear weapons testing might be considered for two reasons. From 1951 until the launch of Sputnik in 1957, at least 124 above-ground nuclear tests were conducted by the United States (U.S.), Soviet Union, and Great Britain. In some circumstances, nuclear radiation is known to cause a visible glow (i.e., Cherenkov radiation)^5^. This phenomenon can be observed in the atmosphere in response to high energy particles (e.g., gamma rays), although it is influenced by both particle energies and atmospheric density^6^. Consistent with this concept, glowing “fireballs” in the sky were reported in multiple instances to occur shortly after nuclear tests in locations where significant nuclear fallout was expected^7,8^. Based on such observations, we hypothesize that some transients might represent an unrecognized atmospheric effect of nuclear testing. Alternatively, it is also possible that fallout from nuclear testing may itself cause direct contamination of astronomical photographic plates, with a characteristic appearance of fogged spots noted on X-Ray sensitive photographic film^9^. We also considered a very different potential reason for links between nuclear testing and transients. Contemporaneous newspaper accounts and records from the Air Force’s Project Blue Book investigation of what are now called Unidentified Anomalous Phenomena (UAP) indicate that unusual, apparently metallic objects of unknown origin were reported in the sky on multiple occasions on dates immediately before, during, and after nuclear weapons tests^7^. UAP have often been reported at nuclear power plants and sites involved in nuclear weapons production as well^7,10^. We hypothesized that if UAP seen during nuclear tests were metallic, they might reflect sunlight (or possibly emit light directly) and thus appear as transients if they were in geosynchronous orbits immediately before or after their appearance during nuclear testing.

**Fig. 1:**
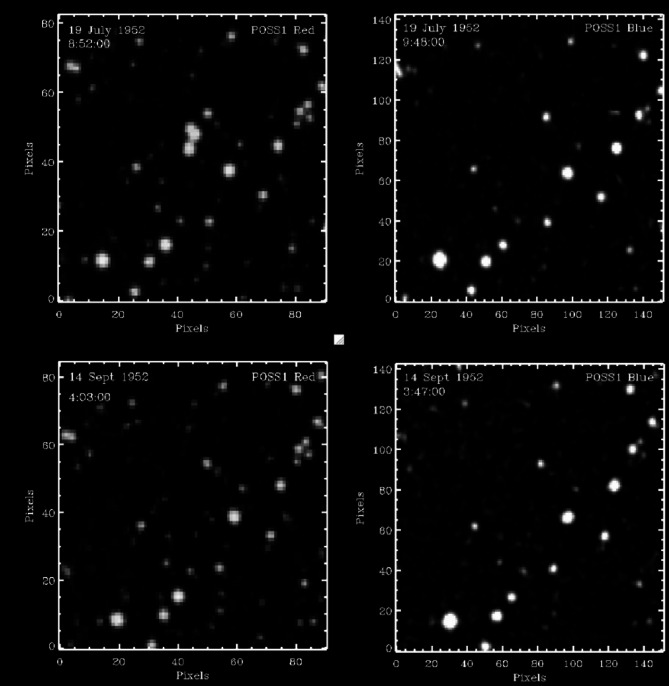
Four exposures of the 3 × 3 arcmin region of sky centered on the triple transient identified in July 1952. Upper left: The POSS I red image on July 19, 1952 at 8:52 (UT) containing the triple transient just above center. Upper right: A 10 m exposure POSS I blue image of the same region taken immediately afterward with no evidence of the triple transient. Lower left and right: POSS I red (left) and blue (right) images taken two months later (September 14, 1952) showing the transient still gone. Adapted from Solano et al. (2024)^4^.

In an extension of this latter hypothesis, transients might also be associated with witness reports of UAP more broadly, outside of the nuclear testing context. Consistent with this, we note POSS-I images from July 19, 1952 and July 27, 1952, each of which exhibit multiple bright transients (see Fig. 1)^4,11^. These dates coincide with two consecutive weekends during which multiple UAP were observed for several hours both visually and on radar over Washington, D.C.^11,12^. We speculate that some transients could potentially be UAP in Earth orbit that, if descending into the atmosphere, might provide the stimulus for some UAP sightings.

In the current study, we conducted a preliminary test of the speculative hypotheses above using a database we have created of > 100,000 transients identified in POSS-I survey images (see Methods). Each of these transients does not appear in a POSS-I image taken shortly before or in images from subsequent surveys. We examined associations of both the presence of any transient (Yes/No) and the number of transients (across the entire sky) identified on each date with: 1) dates of above-ground nuclear testing (from publicly available sources) and 2) reports of at least one UAP on that date (Yes/No) and the total number of independent UAP reported on that date in a comprehensive database of UAP witness reports (UFOCAT; see Methods). While we anticipated significant noise in the UAP sighting data (e.g., due to witness error) and potentially in the transient data as well (e.g., misidentifications related to dust, cosmic radiation, etc.), we believed it was important to subject these novel hypotheses to direct empirical test to provide a preliminary evaluation of possible associations between observed transients and both nuclear testing and UAP sightings.

## Results

### Descriptive characteristics

Transient data were available for the period November 19, 1949 – April 28, 1957, inclusive, with the latter date more than 5 months prior to the launch of the first artificial satellite (Sputnik). Of the 2,718 days in this period, transients were observed on 310 days (11.4%). In the overall sample, the number of transients per date ranged from 0 to 4,528 (across multiple locations on multiple plates), with 5% trimmed mean = 10.09 and median = 0.0. The distribution of number of transients per date was highly right-skewed (skewness = 10.35) and over-dispersed (variance = 28,938.64).

Above-ground nuclear weapons tests (U.S., Soviet, and British) were conducted on 124 days (4.6%) during the study period. UAP reports were recorded in the UFOCAT database on 2,428 days during the study period (89.3%). For days on which at least one UAP sighting was reported, the 5% trimmed mean number of independent sightings (i.e., in different states or countries) was 3.77, with a median of 3.0 sighting reports. The number of UAP reports was significantly higher within a nuclear testing window (5% trimmed mean = 3.68) than outside of a nuclear testing window (5% trimmed mean = 3.31; Mann–Whitney U = 447,057, p = 0.008), suggesting some degree of association between these two outcomes.

### Association of transients with nuclear weapons testing

We first tested for possible associations between occurrence of transients and nuclear weapons tests. The primary nuclear testing outcome reflected a window comprising the test date + /- 1 day (see Methods). Potential associations with transients were tested in two ways. Table 1 displays a 2 X 2 crosstabulation portraying whether each date was within a nuclear testing window (Yes/No) by whether any transient was observed on that date (Yes/No). Transients occurred significantly more often within a nuclear testing window than outside of a nuclear testing window, Chi-Square (1) = 6.94, *p* = 0.008. We note that 15.6% of nuclear test dates were associated with at least one transient whereas only 10.8% of dates outside of a nuclear testing window were associated with a transient. Our findings indicated that the relative risk ratio for a transient to occur when within a nuclear test window (relative to being outside of a nuclear test window) was 1.45 (95% Confidence Interval: 1.10 – 1.90). Thus, a transient was 45% more likely to be observed on dates within a nuclear test window (day of test + /- 1 day) compared to dates outside of a nuclear test window.

**Table 1:** 2 X 2 crosstabulation of transient status on a given date by whether that date fell within a nuclear testing window (test date + /- 1 day). Frequency (and percentage across nuclear testing window categories) are presented. Differences across cells are significant (*p* = .008).

	Transient observed?	
Within a nuclear testing window?	No	Yes
No	2,116 (89.2%)	255 (10.8%)
Yes	293 (84.4%)	54 (15.6%)

Follow-up secondary analyses were then conducted to examine in more granular fashion the timing of the association between nuclear testing and occurrence of transients. Table 2 summarizes the association between occurrence of transients and different time windows relative to nuclear testing, ranging from 2 days before a test until 2 days after a test. The only association that reached statistical significance was for the association in which transients occur 1 day *after* nuclear testing. Transients were observed on 18.5% of days that were 1 day following a nuclear test, whereas transients were noted on only 11.0% of days not meeting this criterion. These findings indicate that the chances of observing a transient were 68% higher on the day following a nuclear test compared to days unassociated with nuclear testing.

**Table 2:** Associations of transients with nuclear testing within different time windows. CI = Confidence Interval.

Time window relative to nuclear test	Percentage of transient positive days within nuclear window	Percentage of transient positive days outside of nuclear window	Chi square value	*P* value	Relative risk (95% CI) of transient occurring within nuclear window
2 days before test	13.7	11.2	0.726	0.394	1.22 (0.775, 1.924)
1 day before test	14.5	11.2	1.307	0.253	1.30 (0.835, 2.017)
Day of test	15.3	11.2	2.016	0.156	1.37 (0.894, 2.102)
1 day after test	18.5	11.0	6.647	0.010	1.68 (1.145, 2.472)
2 days after test	12.9	11.3	0.290	0.590	1.14 (0.712, 1.822)

Beyond dichotomous occurrence of transients, we also tested for differences in the total number of transients observed on a given date as a function of whether that date fell within a nuclear testing window. Significantly more transients were observed on dates within a nuclear testing window (5% trimmed mean = 23.40) than outside of a nuclear testing window (5% trimmed mean = 8.55; Mann–Whitney U = 431,649.5, *p* = 0.007).

### Association of transients with UAP sightings

**Fig. 2:**
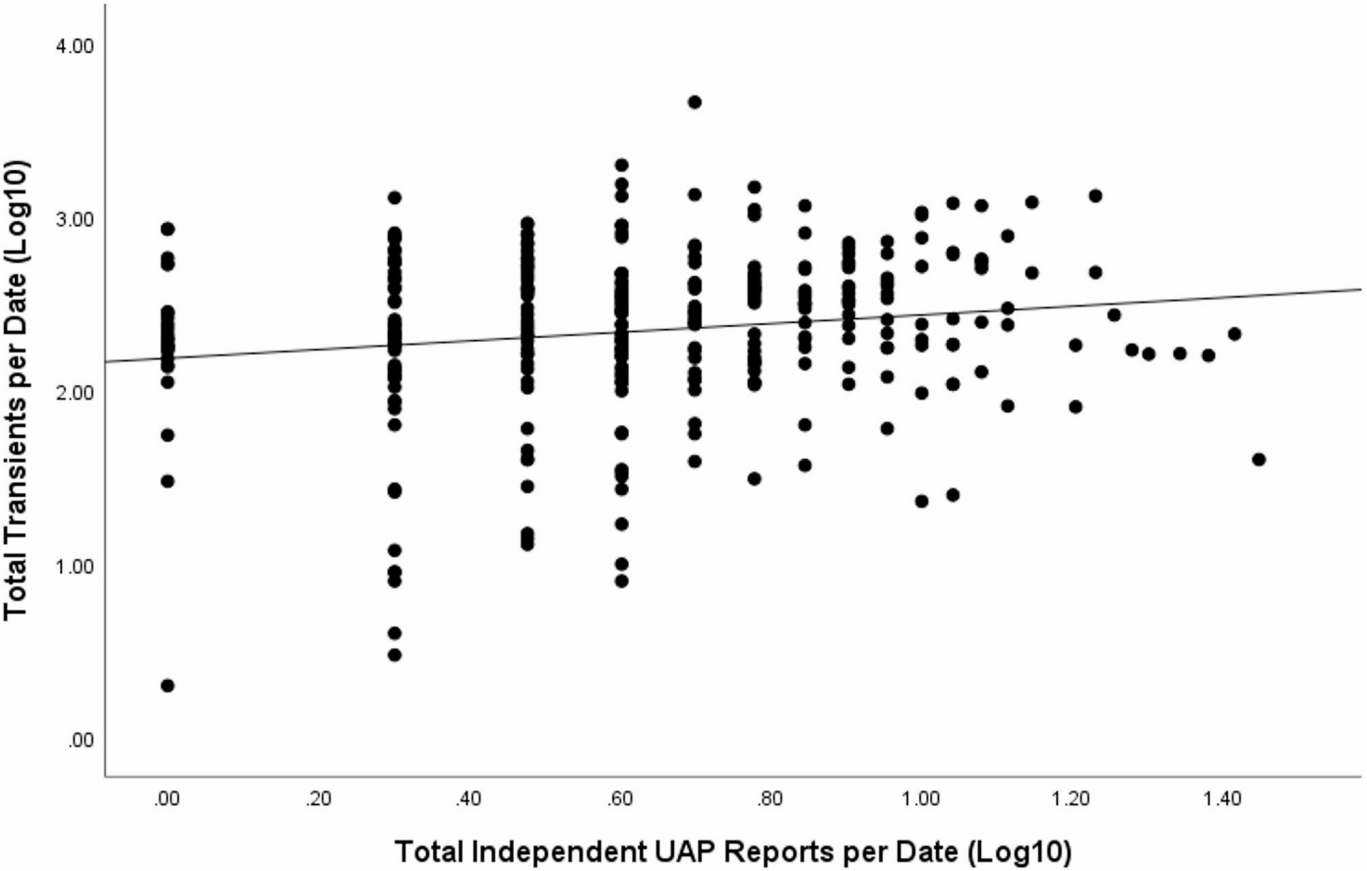
Scatterplot of total number of transients identified by total number of independent UAP reports for dates on which at least one transient occurred (n = 310). Both variables have been log10 transformed to enhance scaling for clarity.

Because UAP reports were so common (at least one report on 89.3% of study dates), examination of possible links between transients and UAP sightings as dichotomous measures was of limited value (this test was not significant; Chi-Square = 2.43, *p* = 0.12). Instead, statistically more powerful analyses based on continuous measures were used to test associations between the *number* of UAP reports and *number* of transients observed on a given date. These analyses employed two approaches. The first approach simply examined the correlation between number of transients and number of UAP sighting reports on a given night. This analysis was restricted to dates on which at least one transient occurred (n = 310), an analysis that eliminates the substantial bias due to the large number of zero values in the transient data (there were no transients observed on 88.5% of the days in the dataset). This simple analysis revealed a very small but statistically significant association (i.e., beyond chance) between the total number of transients and total UAP reports on a given date (Spearman’s rho = 0.138, *p* = 0.015). A scatterplot of this association is presented in Fig. 2.

To address limitations of the simple correlation analysis approach above, we employed a second and statistically more powerful analytic approach to test our transient-UAP hypothesis in a manner that utilized all of the information available in the data. That is, we observed that the total number of transients per date was highly right-skewed and over-dispersed, approximating a negative binomial distribution. We therefore used Generalized Linear Model (GLM) analyses specifying a negative binomial distribution to test associations between number of UAP reports and number of transients each day in the overall sample. Model fit was good (Chi Square = 18.50). Results revealed a significant positive association between number of UAP reported and number of transients observed (Beta = 0.081, Standard Error = 0.006, *p* < 0.001). The exponentiated parameter estimate [Exp(B)] = 1.085] indicated that for every additional UAP reported on a given date, there was an 8.5% increase in the number of transients observed.

Finally, because both nuclear testing and UAP reports were individually associated with transients, we also explored whether their linear combination was associated with total number of transients (i.e., are the observed associations additive?). We created a new categorical variable coded as follows: 0 = No UAP on that date and the date was not within a nuclear testing window, 1 = At least one UAP report that date or the date was within a nuclear testing window, and 2 = At least one UAP report that date and the date was within a nuclear testing window. The dependent variable was total number of transients for each date, so for reasons described above we again used a GLM analysis specifying a negative binomial distribution. Results were statistically significant, Beta = 1.073, Standard Error = 0.0834, *p* < 0.001. Estimated marginal means (with 95% confidence intervals) for each group are presented in Table 3. Dates with no UAP reports that were not within a nuclear testing window were associated with the fewest total transients whereas dates with at least one UAP report and that *were* within a nuclear testing window displayed the highest total number of transients. All pairwise differences between these individual groups were significant (*p*’s < 0.001) and the 95% confidence intervals for each did not overlap. The overall pattern of results suggests that associations of UAP reports and nuclear testing with number of observed transients may be additive.

**Table 3:** Estimated marginal means for total number of transients identified per date across the three combined predictor groups (+ /- UAP reports combined with + /- nuclear testing window). All pairwise comparisons are significant at *p* < .001.

Combined predictor group	Mean total transients	Standard error	95% confidence interval
No UAP reports and not in nuclear window	20.0	1.24	17.69, 22.57
≥ 1 UAP report or within nuclear window	40.6	0.89	38.88, 42.38
≥ 1 UAP report and within nuclear window	58.4	3.25	52.39, 65.15

## Discussion

This study provided a preliminary test of hypothesized associations between short-lived star-like transients identified in POSS-I sky survey images from 1949 and 1957 and both nuclear weapons testing and reports of UAP sightings. The study premise was that identifying contemporaneous correlates of transients might help elucidate their nature and origin, which currently is unknown. Our results revealed several intriguing statistical associations.

First, although not the primary study focus, we observed a small but statistically-significant association between nuclear weapons testing and increased UAP sightings. Significantly more UAP sightings were reported within nuclear weapons testing windows (test date + /- 1 day) than outside of testing windows. To our knowledge, this statistical association has not previously been reported in the peer-reviewed literature, although it is consistent with anecdotal reports of such associations^7^.

Next, in tests of our primary hypotheses, we found that both dichotomous occurrence of transients and the total number of transients observed on a given date were associated with nuclear testing beyond chance. Transients were 45% more likely to be observed on dates that were within a nuclear test window than on dates not in a nuclear test window. More granular examination of the temporal sequencing of these associations revealed that the strongest (and only significant) association was between nuclear testing and increased likelihood of a transient occurring one day after that test.

We also note an intriguing incidental finding regarding possible nuclear testing-transient links. The last date on which a transient was observed within a nuclear testing window in this dataset was March 17, 1956, despite there being an additional 38 above-ground nuclear tests in the subsequent 13 months of the study period. A prior study of associations between UAP reports and nuclear weapons-related production and assembly sites (excluding nuclear weapons tests) concluded that elevated UAP activity at such sites began in 1948, increased dramatically and continued through 1952, but then precipitously decreased in 1953 and remained low through 1975 (end of their study period)^10^. This sudden and sustained decrease in UAP reports at nuclear production facilities in 1953 occurred despite major new nuclear weapons production and assembly facilities coming online during that time (e.g., the Savannah River and Pantex sites)^10^. Taken together, the period between 1953 and 1956 seems to mark a shift in a multiyear pattern of apparent UAP-nuclear associations. While the meaning of these parallel decreases in UAP activity at both nuclear weapons production and testing locations in the mid-1950s is unclear, they may represent convergent evidence for the validity of associations between UAP and nuclear weapons-related activity.

Finally, our hypothesis of associations between transients and UAP reports was also supported. We detected a very small positive correlation, which was well beyond chance, between the number of transients observed and the number of UAP reported on a given date (Spearman’s rho = 0.14). This association was observed when analyses were restricted to dates on which at least one transient occurred, an analysis mitigating the potentially significant bias resulting from the large proportion of dates (88.5%) on which no transients were observed. This finding supports our hypothesis of potentially meaningful associations between transients and UAP reports. Other analyses examining the full sample indicated that for every additional UAP reported on a given date, there was an 8.5% increase in number of transients observed on that date. Overall, findings of this study support our speculative hypotheses that transients exhibit some degree of association with both nuclear testing and reports of UAP. Our results further suggest these associations are additive, with the largest number of transients seen for dates within a nuclear testing window on which at least one UAP was reported.

Our findings do not definitively indicate what transients are nor do they necessarily imply causal associations. However, our results do argue against several prosaic explanations for transients. Our overall pattern of results is clearly not consistent with the proposition that most transients are due to contamination or defects in photographic plates or scanned images, or to any other local confounds at the observatory itself. Contamination of photographic plates by nuclear fallout produces diffuse fogged spots quite different in appearance than the discrete star-like brightness profiles with point spread functions characteristic of transients^3,9^. These explanations would also not account for the association of transients with UAP reports from multiple locations distant from the observatory. Associations between transients and both UAP and nuclear testing reported in this study also cannot be plausibly attributed to any form of observer bias, as the existence of transients was unknown at the time they occurred and the dates/times of nuclear tests were generally unknown to the individuals who were reporting the UAP. Finally, the fact that transients were most likely to occur one day after a nuclear test (rather than the day of the test) argues against bomb debris ejected into the atmosphere as a plausible explanation.

Regarding what transients might be, our findings point toward two hypotheses that could account for associations of transients with both nuclear testing and UAP reports. The first involves an unexpected and previously undocumented atmospheric phenomenon triggered by nuclear detonations or related to nuclear fallout that may serve as a stimulus for some UAP reports and appear as transients on astronomical images. While the latter is potentially plausible, effects in the atmosphere (rather than geosynchronous orbit) would be likely to result in a streak on the image over the 50 min exposure, yet all transients appear as distinct point sources rather than streaks. Moreover, this hypothesis is made even more unlikely given that transients were most often observed one day after a nuclear test; such atmospheric phenomena would have to be sustained and remain localized in one location for approximately 24 h to account for the visual appearance of transients. The second hypothesis is more speculative, drawing on a well-known strand of UAP lore suggesting that nuclear weapons may attract UAP^7,8^. While this alleged connection has been claimed for decades based on anecdotal evidence, it has until now lacked any systematic supporting data. Within this latter hypothesis, our results could be viewed as indicating that transients are artificial, reflective objects either in high-altitude orbits around Earth^13^ or at high altitudes within the atmosphere. Whether and how this hypothesis might be further tested remains to be determined. Regardless of what transients are ultimately determined to be, our results add to growing evidence supporting the interpretation of transients as real observations^1,3,13^ rather than as emulsion defects.

The small magnitude of the significant associations reported must be addressed. Detection of these small effects was enabled by the high statistical power resulting from the large sample size available. Several factors may have contributed to the small magnitude of the associations observed. These associations may have been limited in part by noise in the transient data. Automated methods were applied to identification of the > 100,000 transients comprising the data examined in this study. While a small subset of these have been subjected to manual confirmation, application of more sophisticated systematic validation methods employing artificial intelligence might reduce any misidentifications of transients and result in a higher signal to noise ratio, thereby increasing the magnitude of associations like those reported here. There is also undoubtedly substantial noise in the UAP data examined that could have minimized the size of observed associations. Witness reports are affected by various types of errors^14–16^ and reports in the UFOCAT database that provided UAP data for the current work have not been evaluated for validity in any systematic way. Additionally, the magnitude of the associations between transients and both nuclear tests and UAP might have been limited by the fact that the Palomar Observatory from which transients were observed only provides observations from a single geographic point, whereas nuclear weapons tests and UAP reports can occur worldwide. Finally, transients may be heterogeneous in nature and derived from multiple causes, limiting the magnitude of their association with any single correlate.

In conclusion, data obtained prior to launch of the first artificial satellite in 1957 reveal small but statistically-significant associations between short-lived star-like transients and both above-ground nuclear weapons testing and UAP sightings. Our findings provide additional empirical support for the validity of the UAP phenomenon and its potential connection to nuclear weapons activity, contributing data beyond eyewitness reports. The possibility that some transients may represent UAP events in orbit captured on photographic plates prior to the launch of the first artificial satellite cannot be ruled out. This study adds to the small peer-reviewed literature seeking to apply systematic scientific methods to the study of UAP-related data^8,10,17–20^. The ultimate importance of the associations reported in the current work for enhancing understanding of transients and UAP remains to be determined.

## Methods

### Data sources

#### Transient data

The initial transient dataset consisted of a list of 107,875 transients identified that occurred between 11/19/49 and 4/28/57. These transients were identified in publicly-available scanned images from the POSS-I survey available on the DSS Plate Finder website (https://archive.stsci.edu/cgi-bin/dss_plate_finder). The process used to identify transients and eliminate misidentifications was conducted via an automated workflow detailed fully in Solano et al.^1^. In brief, transients were defined as distinct star-like point sources present in POSS-I E Red images that were absent both in images taken immediately prior to the POSS-I Red image and in all subsequent images. A final criterion for classifying an object as a transient was that there were no counterparts either in PanStarrs DR1 or Gaia DR3 at less than 5 arcsec.

This transient dataset contained the dates, times, and coordinates of each transient identified. For many dates, transients were noted in multiple images reflecting observations of different locations in the sky. The transient dataset (ASCII format) was converted to an SPSS for Windows data file that included a single line for each date on which at least one transient occurred, with a count variable created to summarize the total number of transients observed on each date.

#### Nuclear weapons testing data

An SPSS dataset was created from public sources which included the dates of all above-ground nuclear weapons tests during the study period. Tests conducted by the United States were identified from:

https://nnss.gov/wp-content/uploads/2023/08/DOE_NV-209_Rev16.pdf

Tests conducted by the Soviet Union were identified from: https://en.wikipedia.org/wiki/List_of_nuclear_weapons_tests_of_the_Soviet_Union.

Tests conducted by Great Britain were identified from: https://chrc4veterans.uk/knowledge-hub/british-nuclear-weapons-testing/.

Anecdotal reports from individuals present during nuclear tests in the 1950’s have variously reported UAP to be present at nuclear test sites before, during, and after nuclear tests^7^. Therefore, our primary outcome was a nuclear testing window variable (coded 1/0 for Yes/No) that indicated whether a given date fell within a 3-day window surrounding any nuclear test (test date + /- 1 day). This decision to use a 3-day window as the primary nuclear testing outcome was made while the authors were still blinded to the transient data. To permit subsequent examination of the temporal sequencing of transient associations with nuclear testing at a more granular level, we also created (post-hoc) several variables indicating whether a given date occurred at specific intervals relative to nuclear testing: 2 days before, 1 day before, day of testing, 1 day after, and 2 days after.

#### UAP witness report data

UAP witness report data were derived from the publicly-available comprehensive UFOCAT database maintained by the Center for UFO Studies (https://cufos.org/cufos-publications-databases/ufocat/). This database originated with the U.S. Air Force funded-University of Colorado UFO Study led by Dr. Edward Condon (1966–1968). It has been updated periodically since that time. It represents the most comprehensive publicly-available UAP sighting database covering the 1949–1957 period that was the focus of the current work. The original UFOCAT Microsoft Access database was imported into SPSS. This database contained many identical, duplicate entries (same date and location) obtained from different sources; only a single entry for each discrete report was retained. Next, to reduce the chances of duplicate reports of the same UAP described by separate witnesses on the same date and the same location (i.e., same state), only a single entry was retained in these cases. Finally, a variable reflecting the total count of UAP sightings reported from independent locations on each date was created.

### Procedure

The final analyzed dataset began with creation of an SPSS master file with a separate record for every date within the study period, 11/19/49 to 4/28/57 (n = 2,718 days). Then, the transient database, nuclear test database, and the UAP database were merged by date with this master file. Next, dichotomous variables (coded 1/0 for Yes/No) were created to indicate whether each date in the master file was associated with at least one transient and/or with at least one UAP report. Both dichotomous and continuous variables were available for the transient data (any transient Yes/No and total number of transients identified on each date) and for the UAP data (any UAP Yes/No and total number of independent UAP reports on each date). The nuclear testing variable was only available as a dichotomous index, that is, whether each date fell within a nuclear testing window (coded 1/0 for Yes/No).

#### Statistical analysis

All analyses were carried out using the SPSS for Windows Version 29 statistical package (IBM Corp., Armonk, NY). For testing associations between dichotomous variables [Nuclear Testing Window (Yes/No) versus Transient Observed (Yes/No)], chi-square tests were used. To aid in interpretation of the magnitude of the association between nuclear testing and transients, we adopted a relative risk approach like that commonly used in medical research. That is, we calculated the likelihood of a transient being observed (the “outcome”) based on whether its date was within a nuclear weapons testing window (the “exposure”). This relative risk ratio was calculated using an online calculator: https://www.medcalc.org/calc/relative_risk.php. Due to significantly non-normal distributions of the variables reflecting total number of transients and total number of UAP per night, differences in these variables as a function of nuclear testing were examined using the nonparametric Mann–Whitney U test. For characterizing the nature of group differences in these nonparametric tests, we present 5% trimmed means given the highly skewed distributions of these variables and that median values were generally uninformative (e.g., median total transients = 0). Also for distributional reasons, associations between these two continuous measures were tested using the nonparametric Spearman’s rho correlation. To provide an interpretive context for the magnitude of the association between total number of transients and UAP reported per night, we conducted generalized linear model (GLM) analyses, specifying a negative binomial distribution given the highly right-skewed and over-dispersed nature of the transient data. The resulting exponentiated parameter estimate was then used to derive an estimate of the effect’s magnitude (i.e., impact of number of UAP sightings on total transients observed that date) in terms of incidence rate ratio. For display purposes in Fig. 2, total transients and total UAP reports have both been log10 transformed (after adding a constant [+ 1] to avoid zero values) in order to optimize scaling in the figure.

## Data Availability

The final analyzed SPSS dataset will be made available by the authors upon reasonable request to Dr. Stephen Bruehl (stephen.bruehl@vumc.org).
